# Supplementary figures and images for: Evolutionary and functional analysis of the plant-specific NADPH oxidase gene family in Brassica rapa L
Source: R Soc Open Sci. 2019 Feb 27;6(2):181727. doi: 10.1098/rsos.181727 (PMC6408365; doi:10.1098/rsos.181727)

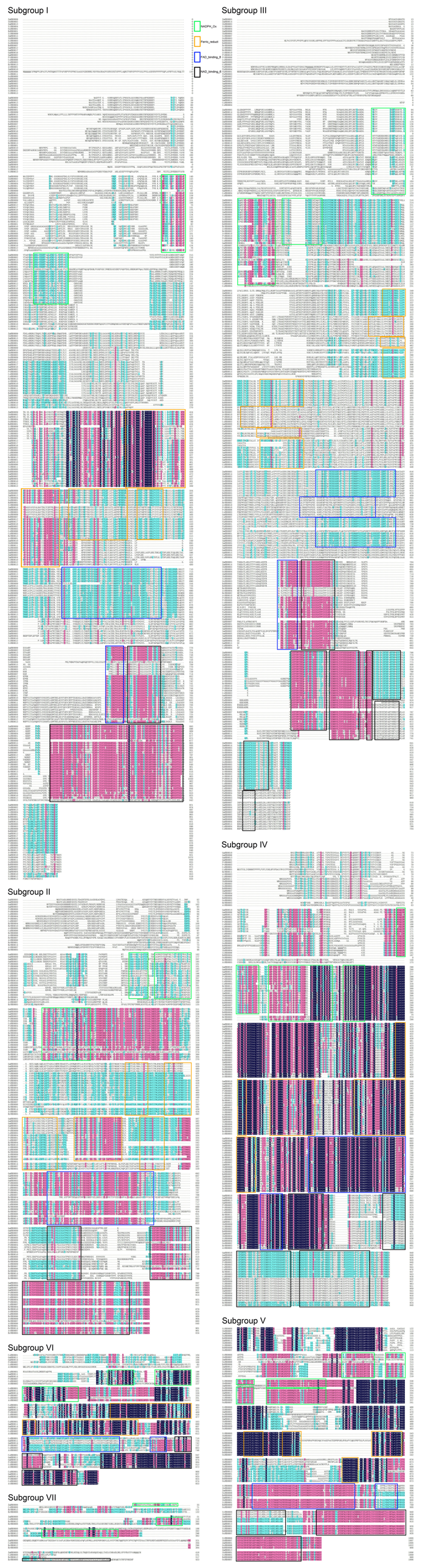

Supplement: Figure S1 [file rsos181727supp1.jpg]

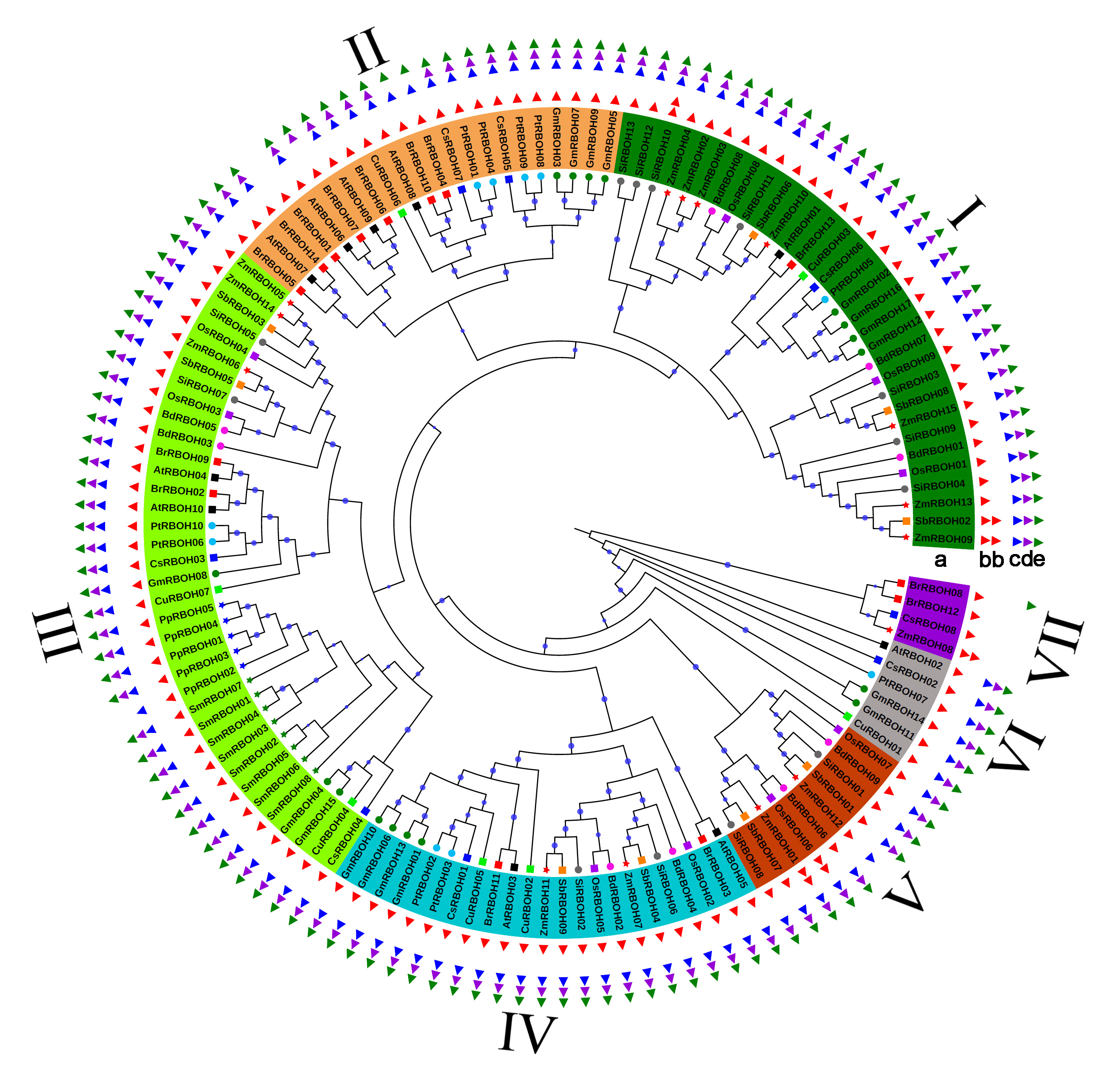

Supplement: Figure S2 [file rsos181727supp2.jpg]

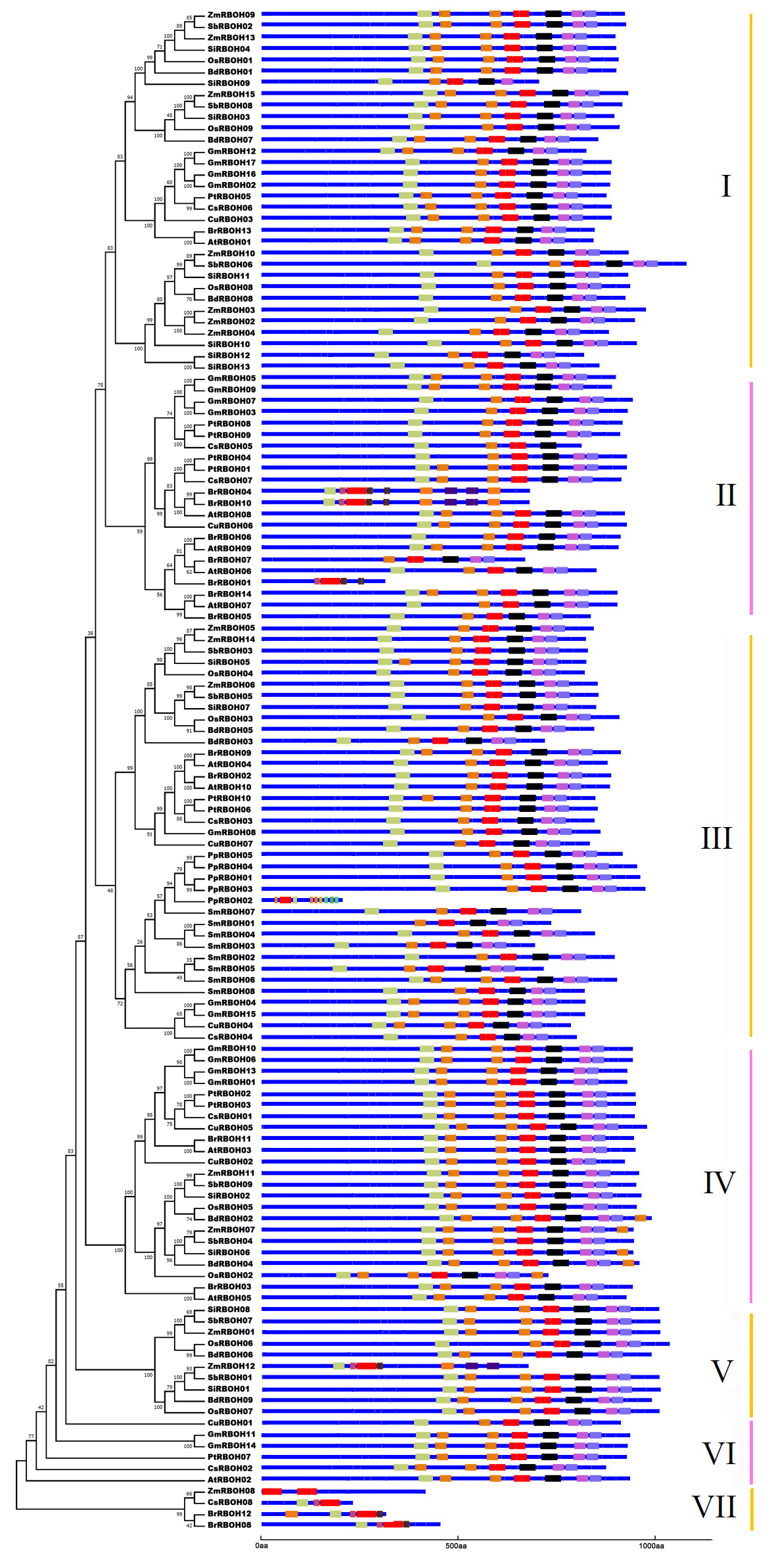

Supplement: Figure S3 [file rsos181727supp3.jpg]

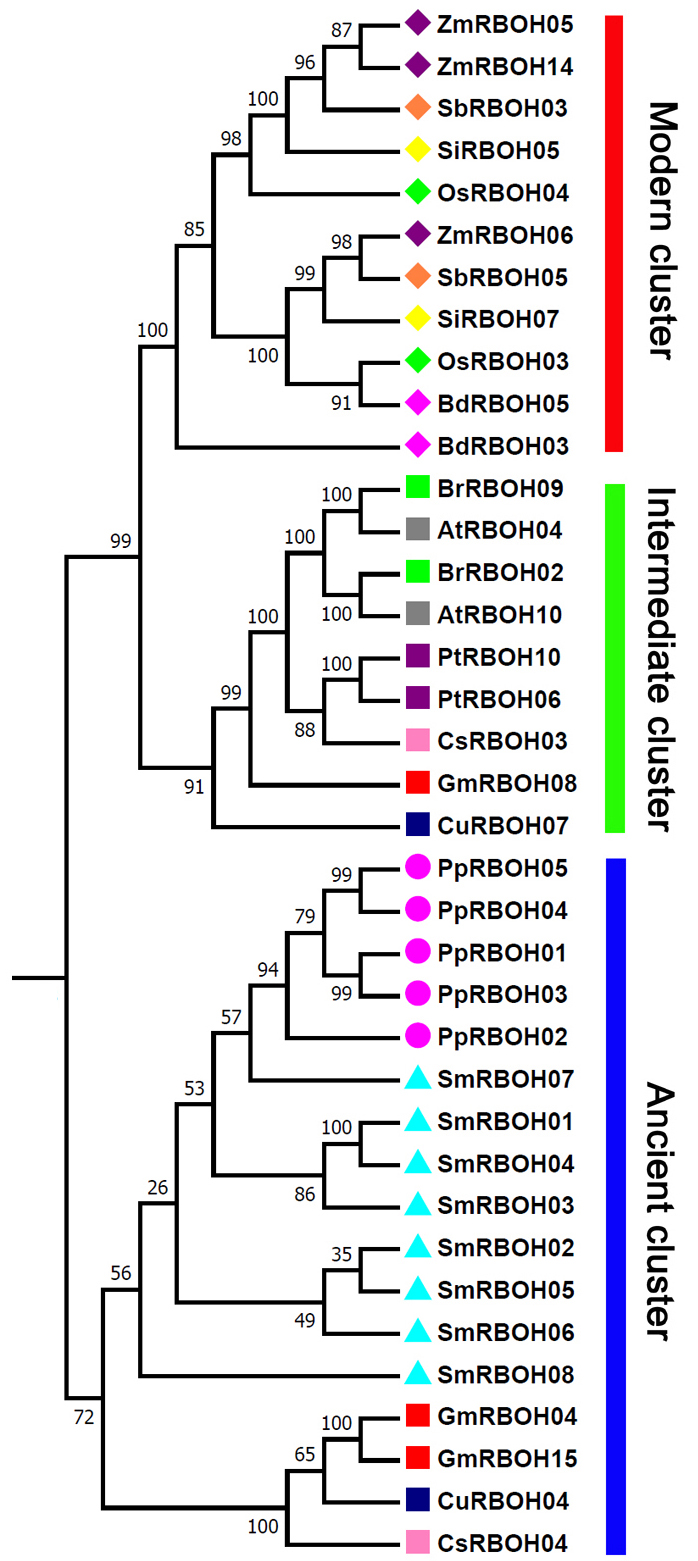

Supplement: Figure S4 [file rsos181727supp4.jpg]

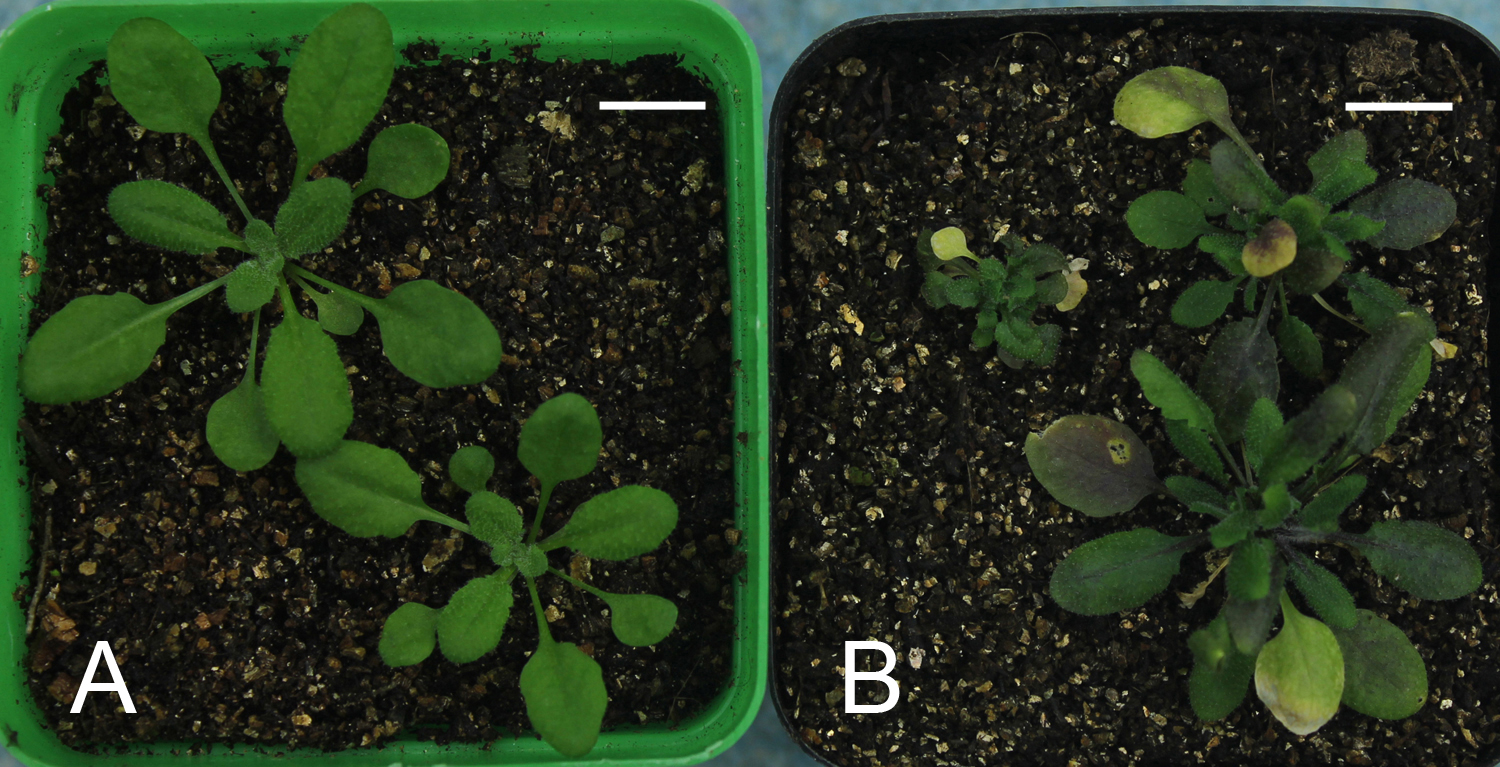

Supplement: Figure S5 [file rsos181727supp5.jpg]
